# Supplementary material for: OPTIMAL: An OPTimized Imaging Mass cytometry AnaLysis framework for benchmarking segmentation and data exploration
Source: Cytometry A. 2023 Oct 5;105(1):36–53. doi: 10.1002/cyto.a.24803 (PMC10952805; doi:10.1002/cyto.a.24803)
Supplement: Supplementary file 1 — Data S1. MIFlowCyt item checklist. [file CYTO-105-36-s001.doc]

**Cytometry Part A**

**Author Checklist: MIFlowCyt-Compliant Items**

| **Requirement** | **Please Include Requested Information** |
| --- | --- |
| 1.1. Purpose | To build a framework for conducting and evaluating/benchmarking the analysis of Imaging Mass Cytometry (IMC) data |
| 1.2. Keywords | Multiplexed imaging, tissue segmentation, image analysis |
| 1.3. Experiment variables | 1. Single cell segmentation of tissues. 2. Optimization of arcsinh cofactor values for data transformation. 3. Batch effect correction by Z-score normalization 4. Choice of dimensionality reduction approach 5. Choice of clustering algorithm 6. Spatial neighborhood analysis method |
| 1.4. Organization name and address | University of Newcastle. Faculty of Medical Sciences, Framlington Place, Newcastle upon Tyne, NE2 4HH, UK |
| 1.5. Primary contact name and email address | Andrew Filby [andrew.filby@ncl.ac.uk](mailto:andrew.filby@ncl.ac.uk)  George Merces [George.merces@ncl.ac.uk](mailto:George.merces@ncl.ac.uk) |
| 1.6. Date or time period of experiment | From 2020 to 2021 |
| 1.7. Conclusions | See main paper for conclusions. |
| 1.8. Quality control measures | The Helios (Hyperion) systems were rigorously maintained and QC’d across all runs. We also used TMAs from the same tonsil tissue (patient) for each separate run as well as the same validated set (panel) of metal conjugated antibodies stained by the same person using a standard approach. We also sought expert pathologist advice about the anatomical and cellular nature of the tonsil tissue to help create “ground truth”. |
| 2.1.1.1. (2.1.2.1., 2.1.3.1.) Sample description | Formalin Fixed Paraffin-Embedded (FFPE) Human Tonsil tissue |
| 2.1.1.2. Biological sample source description | Human tonsillectomy |
| 2.1.1.3. Biological sample source organism description | Human |
| 2.1.2.2. Environmental sample location | Tonsil tissue was placed in to a tissue microarray (TMA) format and stored as a wax embedded block. It was then sectioned to 5 µm depth and mounted on super frost slides |
| 2.3. Sample treatment description | The TMA tissue was subject to an optimized ph9 Tris-EDTA Heat |
| 2.4. Fluorescence reagent(s) description | N/A. But we did use a panel of metal tagged antibodies as shown in supplemental table 1. |
| 3.1. Instrument manufacturer | Fluidigm, now Standard Biotools |
| 3.2. Instrument model | Hyperion (generation one) coupled to a Helios system |
| 3.3. Instrument configuration and settings | Standard |
| 4.1. List-mode data files | All raw images and analysis scripts have been posted here:  <https://www.ebi.ac.uk/biostudies/studies/S-BSST1047> |
| 4.2. Compensation description | Compensation (of sorts) was performed on the IMC data using the approach outlined in the main methods and supplemental methods section. |
| 4.3. Data transformation details | Data was transformed using the arcsinh argument with a range of different cofactor values in order to test for “best resolution” of signal prior to clustering and visualization. |
| 4.4.1. Gate description | Gates were set using FCS express software to denote batch membership of the cells in the total data set (12 batches in all and ~100,000 single cell). |
| 4.4.2. Gate statistics | N/A |
| 4.4.3. Gate boundaries | N/A |

**Notes**

Feel free to use more space than allocated.

You can embed graphics/figures in this document, if needed.

Please make sure to save the document in Microsoft Word version 2003 or older, before uploading to ScholarOne Manuscripts. When uploading this checklist to ScholarOne Manuscripts, please choose the “Supplementary Material for Review” category.

Please note that if your paper is accepted, the checklist will be published as an Online Supporting Information.

For any questions, please contact the Cytometry Part A editorial office at [Cytometrya@wiley.com](mailto:Cytometrya@wiley.com).
